# Interstitial Photoacoustic Sensor for the Measurement of Tissue Temperature during Interstitial Laser Phototherapy

**DOI:** 10.3390/s150305583

**Published:** 2015-03-06

**Authors:** Zhifang Li, Haiyu Chen, Feifan Zhou, Hui Li, Wei R. Chen

**Affiliations:** 1 Fujian Provincial Key Laboratory of Photonic Technology, Key Laboratory of Optoelectronic Science and Technology for Medicine, Ministry of Education, College of Photonic and Electronic Engineering, Fujian Normal University, Fuzhou 350007, China; 2 Department of Cardiovascular Surgery, Fujian Provincial Hospital, Fujian Medical University, Fuzhou 350000, China; E-Mail: chenhaiyulove2002@aliyun.com; 3 Biophotonics Research Laboratory, Center for Interdisciplinary Biomedical Education and Research, University of Central Oklahoma, Edmond, OK 73034, USA; E-Mail: fzhou2@uco.edu

**Keywords:** interstitial photoacoustic signal, temperature sensor, photothermal effect, laser immunotherapy

## Abstract

Photothermal therapy is an effective means to induce tumor cell death, since tumor tissue is more sensitive to temperature increases than normal tissue. Biological responses depend on tissue temperature; target tissue temperature needs to be precisely measured and controlled to achieve desired thermal effects. In this work, a unique photoacoustic (PA) sensor is proposed for temperature measurement during interstitial laser phototherapy. A continuous-wave laser light and a pulsed laser light, for photothermal irradiation and photoacoustic temperature measurement, respectively, were delivered to the target tissue through a fiber coupler. During laser irradiation, the PA amplitude was measured. The Grüneisen parameter and the bioheat equation were used to determine the temperature in strategic positions in the target tissue. Our results demonstrate that the interstitial PA amplitude is a linear function of temperature in the range of 22 to 55 °C, as confirmed by thermocouple measurement. Furthermore, by choosing appropriate laser parameters, the maximum temperature surrounding the active diffuse fiber tip in tissue can be controlled in the range of 41 to 55 °C. Thus, this sensor could potentially be used for fast, accurate, and convenient three-dimensional temperature measurement, and for real-time feedback and control of interstitial laser phototherapy in cancer treatment.

## 1. Introduction

The laser-photothermal effect is a successful approach to tumor destruction since tumor tissue is more sensitive to temperature increases than normal tissue [1]. In the past selective photothermal therapy using an *in situ* light-absorbing agent [2,3] or metal nanoparticles has been developed. While photothermal interaction leads to acute, large-scale and controllable tumor destruction, its long-term effects were rather limited, particularly when treating metastatic cancers [2,3,4,5]. However, laser-tissue thermal interaction can be used in combination with immunotherapy, as thermally destroyed tumor cells could serve as sources of tumor antigens, priming the host immune system. Laser immunotherapy (LIT) [6] was developed as a synergistic approach to treat cancer systemically, through both local laser irradiation and immunological stimulation. LIT has shown great potential in treating late-stage, metastatic cancers, both in preclinical studies [7,8,9,10,11] and in preliminary clinical trials [12,13]. Interstitial laser immunotherapy (ILIT), using a fiber with a cylindrical active diffuse lens, can be an attractive alternative approach to overcome the challenges of non-invasive selective photothermal therapy, particularly when facing deep-seated tumors and highly pigmented skins.

The immunomodulatory effects of thermal interaction have been categorized in three different temperature ranges: fever range (39–40 °C), heat shock range (41–43 °C) and cytotoxic range (>43 °C) [14]. In the first two ranges, the thermal effect modifies both tumor cells and immune cells to stimulate endogenous tumor-specific immune response. In the third range, high temperatures can lead to direct ablation of tumor cells, releasing a large load of tumor antigens; these are taken up by antigen-presenting cells, particularly dendritic cells, and delivered to lymph nodes where they induce an antitumor immune response [14]. The optimal outcome of photothermal tissue interaction, therefore, is to destroy as many target tumor cells as possible, while preserving tumor proteins to be recognized by the host immune system. Laser immunotherapy can achieve a maximum temperature of up to 60 to 70 °C [15], well within the cytotoxic range. At these temperatures, cell death occurs through coagulation necrosis (e.g., at 56 °C with a short, sufficient exposure time) [16]. We therefore aim to keep the maximum temperature in target tissue during interstitial laser irradiation at 56 °C.

Since temperature plays an important role in laser phototherapy, accurate temperature measurement and control are crucial. Current non-invasive methods in tissue temperature measurement include infrared thermography [17,18,19], ultrasound imaging [20], and magnetic resonance thermometry (MRT) [21,22,23]. Infrared thermography can provide sensitive, real-time detection; however, this can measure surface temperature only. Ultrasound can reach deep tissues, but it has relatively low sensitivity and accuracy. Temperature measurements, based on water proton resonance frequency (PRF) in MRT, exploit the temperature dependency of the water proton’s chemical shift to determine tissue temperature of each voxel; and this has been used for interstitial laser therapy [21,22]. MRT provides non-invasive three-dimensional temperature distribution with high sensitivity. However, MRT has relatively low temporal resolution, and its cost and complexity severely limit its practical applications.

Previous reports have shown that photoacoustic (PA) imaging can be used for temperature measurements in tissue. PA signal amplitude shows a linear correlation with temperatures in the range of 10 to 55 °C [24,25], just below the desired threshold temperature of biological responses. Therefore, PA could be used for monitoring tissue temperature and controlling laser phototherapy to optimize thermal effects and for modulating immune responses.

In this study, an interstitial PA sensor is developed for the real-time measurement of tissue temperature during interstitial laser phototherapy. This unique sensor converges a continuous-wave laser light and a pulsed laser light through a fiber coupler to a cylindrical diffuse active lens, and delivers the lights to the same target area at the same time. The two laser lights are used for therapeutic photothermal irradiation and photoacoustic temperature measurement, respectively. Using this sensor, we can measure the maximum temperature increase in target tissue which surrounds the cylindrical active fiber tip. With the use of a bioheat equation, we can obtain tissue temperature in the region of interest. This study could lead to a unique sensor for guidance and control of laser phototherapy in cancer treatment.

## 2. Materials and Methods

### 2.1. Experimental Setup

**Figure 1:**
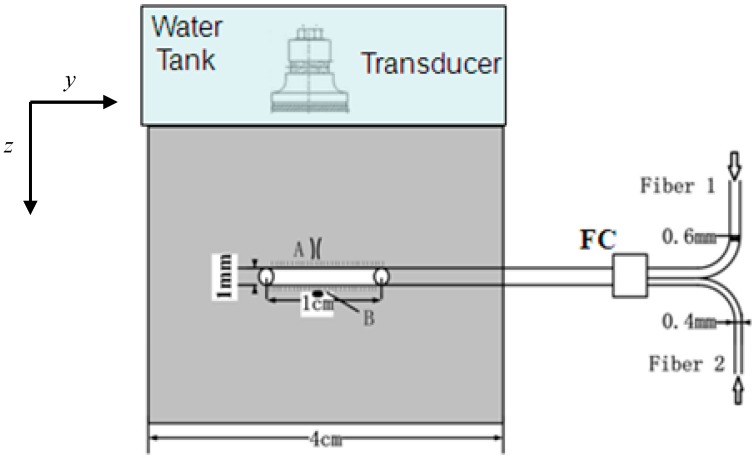
A fiber with a 1.0-cm active tip was placed in the center of the tissue. Both pulse laser light and continuous-wave laser light were directed through two different fibers (1 and 2) into the same active tip (1-cm length and 1-mm diameter) in the tissue through a fiber coupler. The photoacoustic signals at position *A* were detected for temperature determination. A thermocouple needle probe was placed at position *B*, symmetric to position *A*, to calibrate the PA measurement of tissue temperature. A water tank was placed on top of the tissue to enhance the acoustics coupling.

*Ex vivo* pig liver was used for this experiment. The size of the tissue was 4 cm × 4 cm × 1 cm. The pig liver tissue was placed at room temperature (22 °C) prior to the experiment. An optical fiber with a 1.0-cm cylindrical diffuse active tip was placed in the center of the tissue, as shown in Figure 1. Two laser beams were merged through a fiber coupler with a core diameter of 1000 μm. The PA signal at position *A* (0.2 mm from the surface of the active tip) was detected by a focusing transducer. A thermocouple with a core diameter of 0.3 mm was placed at position *B* (symmetric to position *A*), for temperature validation, as shown in Figure 1.

A Nd:YAG laser (Surelite I-10, Continuum, West Newton, MA, USA) with OPO oscillator (Surelite OPO plus, Continuum) was used for photoacoustic wave generation with a wavelength of 810 nm, a repetition frequency of 10 Hz, a pulse width of 6 ns, and output energy of ~6 mJ. The light beam from OPO was divided into two beams using a splitter mirror. One beam was received by a photodiode and displayed on an oscilloscope for calibration, while the other was delivered to the biological sample by Fiber 1 using a core diameter of 600 μm to generate the PA signal. A diode laser (ML-4030D, MIRACLE LASER, Wuhan, China) (wavelength 810 nm), power range 1~30 W, was delivered through Fiber 2 (Figure 1) with a core diameter of 400 μm providing the photothermal effect. Several laser powers, 0.63, 0.93, and 1.24 W, were selected for thermal irradiation. The two optical fibers were placed in parallel; light beams from two fibers were converged through a coupler to the same fiber (core diameter of 1000 μm) with the diffuse active tip.

The upper surface of the tissue sample was coupled by plastic wrap to a water tank, reducing sound attenuation between different media. The depth-resolved photoacoustic signals from the sample were collected by a focused ultrasound transducer (V381, Panametrics, Hamburg, Germany), with a center frequency at 3.5 MHz. The signals were then transferred to an ultrasonic receiver (5800 R, Parametric-NDT, Hamburg, Germany) for amplitude filtering and amplification. Finally, the signals were displayed on a digital oscilloscope (TDS3054C, Tektronix, Johnston, OH, USA). In order to improve the signal-to-noise ratio, the signals on the digital oscilloscope were averaged 8 times and then saved for follow-up data processing. A step motor (SC300-2B, Zolix, Beijin, China) drove the electronic translation machine (TSA200-B, Zolix) for 2D scanning and provided accurate control of the sample platform. The axial and lateral spatial resolution of the PA imaging were 0.3 and 2 mm, respectively, as described previously [26].

### 2.2. Theoretical Foundation of PA Measurement of Temperature 

The imaging method based on the photoacoustic effect uses a short pulse laser to illuminate absorbers in tissue to generate acoustic waves. The measured PA pressure that satisfies the temporal stress confinement is given by [27,28]:

(1) $$P(z)=\text{Γ}{\text{μ}}_{a}F(z,{\text{μ}}_{a},{\text{μ}}_{s},g)$$

where $\text{Γ}=\text{β}c^{2}/C_{p}$ is Grüneisen parameter, β is the expansion coefficient, *c* is the speed of sound, *C_p_* is the specific heat, $F(z,{\text{μ}}_{a},{\text{μ}}_{s},g)$ is the local optical fluence, *z* is the one-dimensional incident depth, ${\text{μ}}_{a}$ is the absorption coefficient, ${\text{μ}}_{s}$ is the scattering coefficient, and g is the anisotropic factor. The Grüneisen parameter Γ is linearly proportional to temperature *T* in the range of 10 to 55 °C for soft tissues [24,25],

(2) Γ=A+BT

where *A* and *B* are constants. Thus, Equation (1) can be further written as:

(3) $$P(z)=(A+BT){\text{μ}}_{a}F(z,{\text{μ}}_{a},{\text{μ}}_{s},g)$$

During interstitial laser phototherapy, the temperature T changes as a function of both irradiation time and laser power. The light emitted from an interstitial fiber was modeled as an isotropically diffusive regime, and the local optical fluence can be expressed as $F(z,{\text{μ}}_{a},{\text{μ}}_{s},g)\propto \text{exp}(-{\text{μ}}_{eff}z)$ [13], where ${\text{μ}}_{eff}=\sqrt{3{\text{μ}}_{a}[{\text{μ}}_{a}+{\text{μ}}_{s}(1-g)]}$ is the effective attenuation coefficient. Based on the values of the absorption coefficient, scattering coefficient, and anisotropic factor of liver tissue (0.73, 0.55 and 0.93 cm^−1^, respectively) at the wavelength of 810 nm [29], we found that ${\text{μ}}_{eff}=3.2$ cm^−1^. The detected superficial PA signal from position A (0.2 mm from the surface of the active tip) was approximated as the superposition of the PA signal *P*(*z*) from a depth of 0.05 to 0.35 mm, since the axial spatial resolution of PA imaging was 0.3 mm. Tissue temperature *T* increases with interstitial irradiation using cylindrical diffusion light. The light diffusion approximation is given by [30]:

(4) $$-D\nabla^{2}\text{φ}\left(r\right)+{\text{μ}}_{a}\text{φ}\left(r\right)=s\left(r\right)$$

where ϕ is the light power density (W·cm^−2^), *D* is the diffusion coefficient (cm^−1^), µ*_eff_ =* (µ*_s_*/*D*)^1/2^, and *s* is the source term (W·cm^−3^). The solution to Equation (4) for an isotropic point light source with power *P*_0_ within an infinite homogeneous medium can be expressed as [30]:

(5) $$\text{φ}\left(r\right)=\frac{P_{0}\text{exp}\left(-{\text{μ}}_{eff}r\right)}{4\pi Dr}$$

The deposited light power *S* can then be determined as *S =* µ*_a_*ϕ(*r*)*.* The Pennes bioheat equation can be used to describe the steady-state temperature fields of tissue irradiated by laser light using an interstitial diffuse fiber; this is modeled as multiple isotropically radiating point sources distributed along the fiber tip with an interval of 1 mm [30]:

(6) $$\text{ρ}\cdot c\cdot \frac{\partial T\left(r,t\right)}{\partial t}=\nabla \cdot \left[k\cdot \nabla T\left(r,t\right)\right]+\sum S\left(r,t\right)$$

where ρ is the density of tissue (kg·cm−3), c is specific heat of tissue (J·Kg^−1^·°C^−1^), k is the thermal conductivity of tissue (W·cm^−1^·°C^−1^), r is the position vector (cm), t is the time (s), and S is the deposited light power (W·cm^−3^). Using Equation (4), tissue temperature can be determined using a simulation program, COMSOL MULTIPHYSICS 5.0. The optical and thermal properties used in Equations (4)–(6) for the simulation are given in Table 1 and Table 2 for liver tissue.

**Table 1:** Optical properties variables for live tissue [29].

Anisotropic Factor (*g*)	Scattering Coefficient (µ*_s_)*	Absorption Coefficient (µ*_a_*)
0.93	5.6 mm^−1^	0.73 mm^−1^

**Table 2:** Thermal and physiological properties of liver tissue [31].

ρ (g/cm^3^)	*c* (J·g^−1^·°C^−1^)	*k* (W·cm^−1^·°C^−1^)
1.05	3.59	0.00566

## 3. Results and Discussion

**Figure 2:**
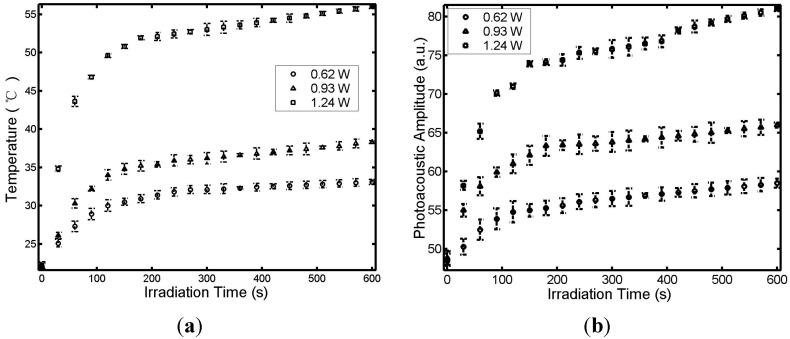
Temperature *T* measured by thermocouple (**a**) and the photoacoustic amplitude measured by the sensor (**b**), at positions *B* and *A*, respectively (see Figure 1), under interstitial laser irradiation of different powers.

**Figure 3:**
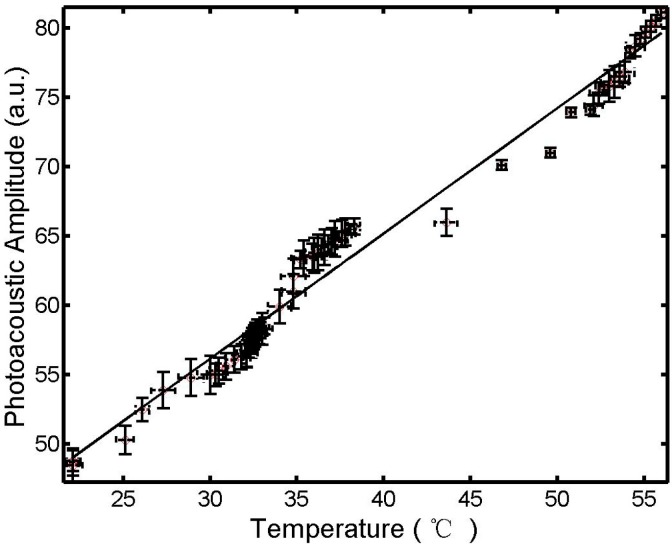
Relationship between photoacoustic signal at position *A* and temperature at position *B* (see Figure 1). The straight line is a regression curve at 95% confidence (*r*^2^ > 0.94 and *p*-value < 0.01).

The temperatures measured by thermocouple and photoacoustic amplitudes, as detected by transducer, were simultaneously recorded every 30 s during laser irradiation with different output powers. Figure 2 shows the temperature *T*, measured by the thermocouple at position *B* and PA signal measured by the transducer at position A (Figure 1), as a function of thermal irradiation time *t* and laser power. The tissue temperature and the PA signal increased sharply during the first 60 s and then slowly plateaued. The plateaued temperature increased with the power of the continuous-wave laser as shown in Figure 2a. The linear relationship between the PA signal at point A and the tissue temperature at point B is shown in Figure 3.

The results in Figure 3 are a graphical representation allowing a good regression of p∝a⋅T+b in the range of 22 to 55 °C, where *a* and *b* are 0.9 and 29.1, respectively. The symmetry around the active fiber tip ensures that the temperatures are the same at point A and point B (Figure 1). Thus, we can use the temperature at point B, directly measured by thermocouple, for the temperature at point A for calibration of PA measurement.

Although the principle of the interstitial PA sensor for temperature measurement is the same as that in previous studies [24,25], the uniqueness of this sensor is reflected on the synchronized interstitial PA temperature measurement and laser photothermal phototherapy. This coupled device allows real-time temperature measurement. Furthermore, an online analysis and control system using this sensor provides immediate feedback, so that treatment parameters can be adjusted during cancer treatment to achieve the desired thermal effect.

**Figure 4:**
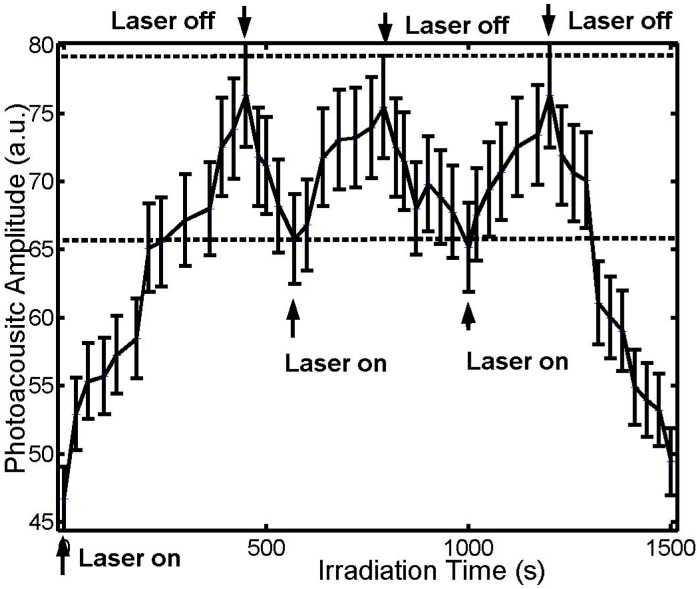
Photoacoustic amplitude *versus* irradiation time. The laser power for thermal irradiation was 1.24 W. The two dotted lines represent the PA amplitudes for the corresponding temperatures at 41 and 55 °C, respectively, based on the linear relationship between PA signals and temperature (Figure 3).

To demonstrate the controlling capability of the unique sensor, we adjusted the thermal radiation power of the laser in an attempt to maintain the tissue temperature at a pre-determined level. When the highest tissue temperature reached 55 °C, the 810-nm thermal laser was turned off, resulting in a temperature decrease. When the highest temperature decreased to 41 °C, the thermal laser was turned on, and the temperature increased. The temperature could therefore be stabilized within the heat shock and cytotoxic range (41 to 55 °C), as shown by the results in Figure 4. Tissue temperature can be similarly controlled within other ranges as well.

**Figure 5:**
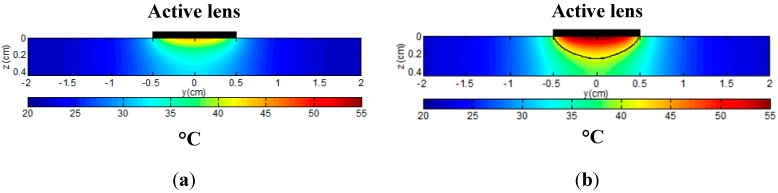
Two-dimensional temperature distribution in tissue. The active diffusion tip is along the y direction, while the z direction is perpendicular to the active tip. (**a**) Temperature distribution with a maximum temperature of 41 °C; (**b**) Temperature distribution with a maximum temperature of 55 °C with the black curve denoting a temperature of 41 °C.

In this study, thermocouple and PA temperature measurement took place at the tissue boundary, close to the surface of the active fiber tip. The temperature distribution in the tissue away from the active fiber tip was simulated using the bioheat equation (Equation (4)). A two-dimensional temperature distribution is given in Figure 5 (due to symmetry, only the lower half of the tissue configuration is shown), with the center of the active lens as the origin. Figure 5a shows the temperature distribution when the maximum target tissue temperature was at 41 °C (when the thermal laser was turned off). During laser irradiation, the maximum tissue temperature increased from 41 to 55 °C, with a large region of the tissue maintaining a temperature above 41 °C, as marked by the black boundary in Figure 5b.

## 4. Conclusions

In this study, an interstitial photoacoustic sensor for monitoring temperature during interstitial laser phototherapy was presented. Our results demonstrate that the PA amplitude is a linear function of the highest temperature surrounding the active fiber tip. Furthermore, the highest temperature can be controlled in the range of 41 to 55 °C. With the confirmation of the linear relationship between the temperature and the PA signal (Figure 2 and Figure 3), we can simulate the tissue temperature using a reliable bioheat equation. Thus, this PA sensor, coupled with an interstitial laser phototherapy device, can be used to control both thermal interactions and induced immunological responses in the treatment of cancers.

The uniqueness of this proposed PA-thermal coupling device is reflected in its simplicity and its effectiveness. The laser beams for treatment and temperature detection were uniquely coupled to irradiate the target tissue with spatial and temporal synchronization. With an online analysis system, it is relatively easy and inexpensive to obtain accurate tissue temperature distribution in the entire region of interest. Another advantage of this proposed system is the real-time feedback and online control of laser photothermal therapy.

This technique certainly has certain limitations. One limitation is posed by the challenges in generating photoacoustic signals, since PA signals are sensitive to boundary conditions and tissue types. Another major challenge stems from the complex nature of tumors (morphology and structure). To improve the effectiveness of this device, light-absorbing agents, such as chemical dyes or nanoparticles, can be used for further enhancement of the selective photothermal effect. While this study focuses on a photoacoustic sensor for monitoring temperature during interstitial laser phototherapy, using an 810-nm pulse laser, other approaches may also be considered in a larger context of temperature determination, using laser light of different wavelengths for better tissue penetration [32].

Tissue characterization for tissue optical properties, prior to the use of this device, will also improve the accuracy of temperature measurement, and will provide optimal control of laser cancer treatment. Although complete solutions addressing all challenges, which consider the irregular vascular structure and unpredictable size of tumors, do not currently exist, this technique will certainly provide a reliable measurement for tissue temperature within the range of biological interest. More importantly, this technique is not designed to achieve a perfect photothermal interaction for cancer treatment, as thermal treatment alone has failed to prove an effective modality. This technique may be used for interstitial laser immunotherapy where desirable thermal effects are the precursor for induced tumor-specific immunity. The photothermal effect is not required to completely destroy the tumor tissue, but only to destroy and interrupt tumors cells at a sufficient level to assist the activation and enhancement of the host immune system. This technique therefore has a high clinical relevance.
